# Effects of Longer Seated Lunch Time on Food Consumption and Waste in Elementary and Middle School–age Children: A Randomized Clinical Trial

**DOI:** 10.1001/jamanetworkopen.2021.14148

**Published:** 2021-06-22

**Authors:** Xanna Burg, Jessica Jarick Metcalfe, Brenna Ellison, Melissa Pflugh Prescott

**Affiliations:** 1 Department of Food Science and Human Nutrition, University of Illinois at Urbana-Champaign, Urbana; 2 Department of Agricultural and Consumer Economics, University of Illinois at Urbana-Champaign, Urbana

## Abstract

**Importance:**

Health experts recommend at least 20 minutes of seated lunch time for children, but no federal policy for lunch period duration exists in the United States. Additional strategies in the National School Lunch Program for mitigating food waste are needed to maintain the viability of the Healthy, Hunger-Free Kids Act.

**Objective:**

To assess the effect of a longer seated lunch time on food consumption and waste among elementary and middle school–age children.

**Design, Setting, and Participants:**

This randomized within-participant crossover trial was conducted from June 3 to June 28, 2019, for a total of 20 study days. All attendees of a summer camp held at the University of Illinois at Urbana-Champaign, were invited to participate in the study. Participants were elementary and middle school–age children and were provided every study day with lunch prepared according to the National School Lunch Program nutrition standards.

**Intervention:**

Five menus were served throughout the study. A 20-minute or 10-minute seated lunch condition was randomly assigned to each day within the 5 menus.

**Main Outcomes and Measures:**

The primary outcomes were food consumption, waste, and dietary intake, which were analyzed separately for each meal component (fruit, vegetable, entree [protein plus grain], beverage [both milk and water], and milk alone). Dietary intake was assessed for calories, fat, carbohydrates, fiber, protein, vitamin D, calcium, iron, and potassium. Participant behaviors were observed during the meal, including seated time as well as level of talking and phone use.

**Results:**

A total of 38 children with 241 lunch trays were observed. The 38 children had a mean (SD) age of 11.86 (1.23) years and 23 were female participants (61%); 30 had a non-Hispanic/non-Latino ethnicity (79%) and 23 were White individuals (61%). During 10 minutes of seated lunch time, participants consumed significantly less fruit (−11.3 percentage points; 95% CI, −18.1 to −4.5) and vegetables (−14.1 percentage points; 95% CI, −22.7 to −5.7) compared with 20 minutes of seated lunch time. Entree and beverage consumption and waste did not differ between the 10-minute and 20-minute seated lunch conditions. Participants also consumed significantly more and wasted significantly less calories (−22.03 kcal; 95% CI, −39.47 to −4.61 kcal), carbohydrates (−3.81 g; 95% CI, −6.20 to −1.42 g), dietary fiber (−0.51 g; 95% CI, −0.81 to −0.19 g), protein (−1.11 g; 95% CI, −2.17 to −0.04 g), iron (−0.20 mg; 95% CI, −0.38 to −0.02 mg), and potassium (−53.49 mg; 95% CI, −84.67 to −22.32 mg) during the 20-minute seated lunch condition.

**Conclusions and Relevance:**

This study found that fruit and vegetable consumption increased in school-age children during a 20-minute seated lunch condition. This finding supports policies that require children to receive at least 20 minutes of seated lunch time; such policies could have favorable implications for children’s dietary intake and food waste.

**Trial Registration:**

ClinicalTrials.gov Identifier: NCT04191291

## Key Points

**Question:**

What is the effect of longer seated lunch time on children’s consumption and waste of lunches that were prepared according to the National School Lunch Program nutrition standards?

**Findings:**

In this crossover trial of 241 lunch observations from 38 children, 20 minutes of seated lunch time were associated with significantly more consumption and significantly less waste of fruits and vegetables compared with 10 minutes of seated lunch time. Entree and beverage consumption was not significantly different between the 10-minute and 20-minute seated lunch conditions.

**Meaning:**

Results from this study support policies that require 20 minutes of seated lunch time for school-age children, which could lead to better diet quality and reduced food waste.

## Teaser

This crossover trial evaluates eating and noneating behaviors of school-age children during 2 different lunch periods (10 minutes vs 20 minutes) in a simulated school cafeteria setting that served meals according to National School Lunch Program nutrition standards.

## Introduction

In the US, studies found that 18.5% of children were obese^1^ and most children 9 years or older did not meet the recommended vegetable or fruit intake.^2^ In addition, fiber, calcium, potassium, and vitamins A, D, E, and C were reported to be underconsumed nutrients in the US population.^3^ The National School Lunch Program (NSLP) reaches 29.6 million children annually^4^ and ensures access to foods that can address common nutrient shortfalls. Previous research demonstrated the potential of strong NSLP nutrition standards to reduce childhood obesity among vulnerable youth.^5^ The Healthy, Hunger-Free Kids Act of 2010 strengthened the nutritional requirements for school meals, providing an opportunity for the NSLP to address childhood obesity and promote health equity across the US.^6^

Multiple studies have found either no substantial change or decreased food waste after the implementation of the Healthy, Hunger-Free Kids Act.^7,8^ Yet, the nutritional requirements for milk, whole grains, and sodium were weakened in 2018, citing concerns about waste.^9^ Given that food waste has been a long-standing unintended consequence of the NSLP that predates the Healthy, Hunger-Free Kids Act,^10^ other factors associated with NSLP food waste, such as the amount of time children have to eat, should be considered.

The American Academy of Pediatrics and the Centers for Disease Control and Prevention recommend that children have at least 20 minutes of seated lunch time, which refers to the amount of time a child can spend eating after sitting down with their meal.^11,12^ Previous research has estimated that a 30-minute lunch period provides enough additional time for traveling and waiting in line to achieve 20 minutes of seated time.^13,14^ In 2016, only half of all US school districts required or recommended 20 minutes of seated lunch time.^15^

The association between seated lunch time and child eating behaviors has been examined using observational study designs. Cohen et al^16^ found that children with less than 20 minutes to eat consumed less of their entree, milk, and vegetable items compared with children who had 25 minutes or more time. Bergman et al^17^ found similar results: 30-minute lunch periods were associated with greater overall food consumption compared with 20-minute periods, decreasing food waste from 43% to 27%. However, these studies did not control for the inherent variation in personal food preferences across different lunch menus. Furthermore, children’s social behaviors and mobile phone use may be associated with the amount of food consumed during lunch.

The current crossover trial aimed to assess the effect of a longer seated lunch time on food consumption and waste among elementary and middle school–age children. We conducted this study in a controlled setting that simulated school cafeterias serving NSLP menus.

## Methods

The University of Illinois at Urbana-Champaign Institutional Review Board approved the study protocol. Children provided written assent, and their parents provided written consent for participation. We followed the Consolidated Standards of Reporting Trials (CONSORT) reporting guideline.

### Study Design, Sample, and Setting

This randomized within-participant crossover trial assessed eating behaviors during varying lengths of seated lunch time in children, age 8 to 14 years, who attended a summer camp at the University of Illinois at Urbana-Champaign. The study took place over 4 consecutive weeks, from June 3 to June 28, 2019, for a total of 20 study days. Children received a free lunch each day that met the NSLP nutrition standards. Five menus were served throughout the study to provide variety, with each menu occurring 4 times over the course of the 20-day trial. We randomly assigned a 10-minute or 20-minute seated lunch condition to each menu, resulting in each menu being served exactly 2 times for both 10-minute and 20-minute seated lunch conditions (Supplement 1).

We invited all summer camp attendees to participate in the study. Parents who consented to their children’s participation provided demographic information, including race/ethnicity, using options that we provided (ie, Hispanic/Latino, Non-Hispanic/non-Latino, American Indian/Alaska Native, Asian, Black or African American, Native Hawaiian or other Pacific Islander, or Other). Race/ethnicity information was collected to understand how representative the study sample was compared with children who consumed NSLP meals during the regular school year. Inclusion criteria were summer camp attendance and consumption of the provided lunch. The summer camp had a separate enrollment with participation that varied each week. Children could enroll for multiple weeks of camp.

Research staff prepared lunch in a commercial kitchen and preportioned servings ahead of participant food selection using standard-sized serving utensils. Each food item’s reference weight was measured before lunch.

Using an offer vs serve format, the staff served those items that children verbally requested. Water and milk (1% plain and 1% chocolate) were the only 2 drink options available. To ensure that NSLP reimbursable meal requirements were met, the staff took a photograph to record each participant’s food selection when they left the lunch line.^18^ Seated lunch time officially began when the last child exited the line and sat down at the table. The staff announced and posted on a sign the exact time that lunch would end and made an additional announcement 5 minutes before the lunch was scheduled to end. Children were not allowed to request additional food items (ie, no second serving) during lunch but did have access to unlimited cups of water.

Research staff collected observational data on the time the child sat down to eat, additional times the child stood up during lunch, social interactions the child had with other children during lunch, and time the child stood up at the end of lunch. Staff collected these data while sitting at separate tables that were interspersed among participant tables and ate the same lunch food to make observation less noticeable.

### Measures

The independent variable, amount of seated lunch time, was assessed in 2 ways. The first measure was a categorical variable representing the randomly assigned length of seated lunch time: 10 or 20 minutes. This measure was a real-world indicator of seated lunch times, knowing that some children may have had more or less than the target 10 or 20 minutes because of other factors such as being late or using the restroom. The second measure was a continuous variable for the number of minutes a child was seated. Seated lunch time was calculated by subtracting the time a participant stood up at the end of lunch from the time a participant sat down at the beginning of lunch to the nearest second, as recorded by the designated staff observer. Any time that a participant left their seat was documented and subtracted from the recorded seated time. This measure was a more precise way to measure seated lunch time, accounting for the variability in the actual amount of time a participant was seated. Interrater reliability assessed from multiple observers for the same participant showed excellent agreement^19^ for the length of lunch time (intraclass correlation coefficient [ICC], 0.998; 95% CI, 0.997-0.999) and nonseated lunch time (ICC, 0.954; 95% CI, 0.888-0.979).

The primary outcomes were food consumption and waste by meal component: entree, fruit, vegetable, beverage (both milk and water), and milk alone. At the end of lunch, research staff weighed the items left on each food tray to the nearest 0.5 grams. Remaining beverage items were measured to the nearest 0.5 millilters using a graduated cylinder. Nutrition Data System for Research software, version 2018 (Nutrition Coordinating Center, University of Minnesota), was used to calculate the calories, total fat, carbohydrates, dietary fiber, protein, vitamin D, calcium, iron, and potassium of each item.^20,21,22^

Research staff rated each participant’s level of talking (ICC, 0.954; 95% CI, 0.932-0.967) and phone use (ICC, 0.967; 95% CI, 0.954-0.976) on a scale of 0 to 5 during the first 5 minutes (10-minute condition) or 15 minutes (20-minute condition) and the last 5 minutes of lunch. The scale for these behaviors was as follows: 0 indicated no talking or phone use; 1, minimal talking or phone use; 2, talking or phone use about 25% of the time; 3, talking or phone use about 50% of the time; 4, talking or phone use about 75% of the time; and 5, almost constant talking or phone use. An overall mean rating for both talking and phone use was calculated using ratings during the first part of lunch and during the last 5 minutes of lunch.

After lunch concluded, each participant completed a 2-question survey to rate how their lunch looked and tasted. Participants used a rating scale with 5 options ranging from “I really didn’t like it” to “I really liked it.” Responses were coded between 1 and 5, with 5 indicating the highest level and 1 the lowest level of liking the look and taste of the food.

### Statistical Analysis

Statistical analyses were conducted with R, version 3.6.2 (R Foundation for Statistical Computing), and the lme4, lmerTest, and emmeans packages.^23,24,25,26^ Two-tailed hypothesis tests and a threshold of 2-sided *P* < .05 for statistical significance were used. The study sample was recruited from a fixed population of children who attended summer camp. Mixed-effects linear regression models assessed all continuous outcome measures and included a random effect for participants to control for repeated measures (Supplement 1). All models included fixed effects for menu (5 levels), previous menu exposure to control for past participation (4 levels), mean talking rating (continuous), and mean phone use rating (continuous). Models that assessed the amount wasted, nutrients consumed, and nutrients wasted included an additional fixed effect for reference weight or reference nutrient to control for the variability of available food or nutrients preconsumption.

The 2 primary outcomes, food consumption and waste, were assessed individually across 5 meal components using the 2 measures of seated lunch time. The categorical condition models assessed the estimated marginal means of each condition and the contrast (10 minutes minus 20 minutes). Models that used continuous seated lunch time assessed the β coefficient and can be interpreted as the change in outcome for a 1-minute increase in seated lunch time. Nutrient models assessed the nutrients found in a participant’s total meal and used only the categorical measure of seated time.

## Results

Throughout the study, 38 of the 53 children who were assessed for eligibility (72%) participated in 1 or more study days (Figure). A total of 241 food trays were collected from 38 children, who participated for a mean number of 6 study days (Figure). The 38 children had a mean (SD) age of 11.86 (1.23) years; 23 were female (61%) and 15 were male (39%) participants, 30 had a non-Hispanic/non-Latino ethnicity (79%), and 23 were White individuals (61%) (Table 1).

**Figure.**
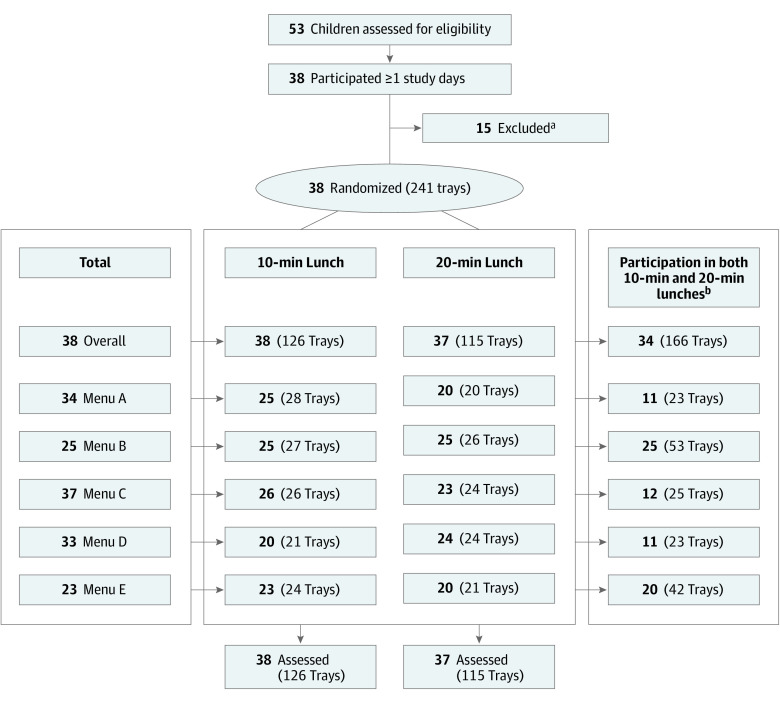
Flow of Participation and the Study Design ^a^Inclusion criteria were attendance in the summer camp and consumption of the prepared lunch. Children were excluded (not counted) if they lacked written parental consent, brought their own lunch, or otherwise refused to eat the lunch at camp. ^b^Children were counted if they participated in both the 10-minute and 20-minute lunch conditions for the same menu. For the overall category, children had to have at least 1 occurrence of a paired 10-minute and 20-minute lunch for 1 of the 5 menus.

**Table 1.** Characteristics of the Lunch Study Participants

Characteristic	No. (%)
Total No. of participants	38
Age, mean (SD), y	11.86 (1.23)
Sex	
Female	23 (61)
Male	15 (39)
Ethnicity	
Hispanic/Latino	2 (5)
Non-Hispanic/non-Latino	30 (79)
Not reported	6 (16)
Race	
White	23 (61)
Black or African American	1 (3)
Asian	3 (8)
Other^a^	1 (3)
≥2 races	5 (13)
Not reported	5 (13)
Most recent grade completed	
2nd Grade	1 (3)
3rd Grade	5 (13)
4th Grade	8 (21)
5th Grade	11 (29)
6th Grade	8 (21)
7th Grade	3 (8)
Not reported	2 (5)
Academic year lunch provision^b^	
Eats school lunch	14 (37)
Brings lunch from home	16 (42)
Other	5 (13)
Not reported	3 (8)
Eligible for free/reduced-price lunch^c^	
Yes	10 (26)
No	23 (61)
I don’t know	2 (5)
Not reported	3 (8)

^a^ Other race was an option for parents to select when completing the demographic form, and additional information was not specified. American Indian/Alaska Native and Native Hawaiian or other Pacific Islander races had zero responses.

^b^ Parents reported how their child ate lunch on most school days during the regular school year. Other included children who, on most days, ate both lunch brought from home and food from the school lunch program.

^c^ Parents reported if their child was eligible for free or reduced-price meals during the most recent school year.

A total of 34 children (89%) had at least 1 pair of 10-minute and 20-minute seated lunch observations for 1 of the 5 menus. Of the 241 trays, 166 (69%) represented pairs of observations that had at least 1 occurrence of both 10-minute and 20-minute conditions for a menu (Figure). About half of the trays (126 [52%]) were from the 10-minute condition. The mean (SD) duration of lunch on days with a 10-minute condition was 11.76 (1.62) minutes, and the mean (SD) duration of lunch on days with a 20-minute condition was 21.59 (1.79) minutes.

Participants left their table for a mean (SD) time of 0.66 (0.66) minutes and had a mean (SD) rating of 3.03 (1.54) for talking and 0.42 (1.10) for phone use during lunch. Participants had higher mean (SD) talking ratings during the 20-minute vs 10-minute seated lunch condition (3.56 [1.46] vs 2.56 [1.45]; *P* < .001). The mean (SD) phone use ratings did not differ by the 20-minute vs 10-minute condition (0.47 [1.15] vs 0.38 [1.06]; *P* = .51). In general, participants had similar mean (SD) ratings for how their lunch looked for both the 20-minute and 10-minute conditions (4.11 [0.84] vs 4.12 [0.83]; *P* = .95) and for how their lunch tasted for both the 20-minute and 10-minute conditions (4.27 [0.96] vs 4.20 [0.88]; *P* = .56), and those ratings did not differ by seated lunch time.

Overall, participant selection of meal components was high. Of the 241 trays the participants selected, 241 trays (100%) contained an entree, 232 (96%) contained a fruit, 210 (87%) contained a vegetable, 238 (99%) contained a beverage (water or milk), and 124 (51%) contained milk. Selection did not differ between the 10-minute or 20-minute condition.

During the 10-minute seated lunch condition, participants consumed significantly less and wasted significantly more fruits and vegetables. Participants consumed 11.3 (95% CI, −18.1 to −4.5) percentage points less fruit and 14.1 (95% CI, −22.7 to −5.7) percentage points less vegetables in the 10-minute than the 20-minute lunch (Table 2). This reduced consumption translated to participants wasting 9.61 (95% CI, 3.62-15.60) more grams of fruit and 8.79 (95% CI, 2.51-15.07) more grams of vegetables during the 10 minutes of seated time compared with 20 minutes. A similar pattern was observed when accounting for seated lunch time in minutes (Table 3). For every 1-minute increase in seated lunch time, participants consumed 1.240 (95% CI, 0.569-1.915) percentage points more of their fruit and 1.685 (95% CI, 0.870-2.502) percentage points more of their vegetable. Consumption and waste of entree, beverage, and milk did not differ by seated time.

**Table 2.** Estimated Means of Food Consumption Between 10-Minute and 20-Minute Seated Lunch Times

Tray item	10-min Lunch, No.	20-min Lunch, No.	Total weight/volume, mean (SD)^c^	% Consumed^a^				Weight/volume consumed^b^			
				10-min Lunch, mean (SE)^d^	20-min Lunch, mean (SE)^d^	Contrast (SE)^e^	Contrast 95% CI	10-min Lunch, mean (SE)^d^	20-min Lunch, mean (SE)^d^	Contrast (SE)^e^	Contrast 95% CI
Entree, g	126	113	133.02 (38.05)	87.4 (5.5)	88.7 (5.4)	−1.3 (2.7)	−6.6 to 4.0	113.50 (7.35)	118.01 (7.20)	−4.51 (3.63)	−11.68 to 2.65
Fruit, g	120	110	93.10 (32.35)	72.9 (7.1)	84.2 (7.0)	−11.3 (3.5)	−18.1 to −4.5	70.63 (6.19)	80.24 (6.08)	−9.61 (3.04)	−15.60 to −3.62
Vegetable, g	113	95	66.02 (20.70)	51.2 (7.8)	65.3 (7.6)	−14.1 (4.3)	−22.7 to −5.7	32.45 (5.74)	41.24 (5.63)	−8.79 (3.18)	−15.07 to −2.51
Beverage, mL^f^	122	113	274.84 (213.11)	71.9 (7.1)	74.7 (6.9)	−2.8 (3.1)	−9.0 to 3.4	203.30 (18.98)	213.36 (18.51)	−10.06 (8.65)	−27.12 to 7.00
Milk, mL	61	60	236.59 (0)	64.3 (9.0)	70.7 (8.7)	−6.4 (3.8)	−14.0 to 1.2	152.18 (21.33)	167.37 (20.54)	−15.19 (9.07)	−33.20 to 2.83

^a^ Models included a random effect for participant and fixed effects for seated lunch time, menu, previous menu exposure, mean talking rating, and mean phone use rating.

^b^ Models included a random effect for participant and fixed effects for seated lunch time, menu, previous menu exposure, mean talking rating, mean phone use rating, and preconsumption reference weight or volume.

^c^ Mean weight in grams (entree, fruit, and vegetable) or mean volume in milliliters (beverage and milk) for the tray item across all lunch times.

^d^ Means reported were adjusted for the covariates in the models.

^e^ Contrast was the result of 10-minute minus 20-minute lunch.

^f^ Beverage category included a selection of milk, water, or both milk and water.

**Table 3.** Food Consumed per Minute of Increased Seated Lunch Time

Tray item	No. of trays	% Consumed^a^		Weight/volume consumed^b^	
		β (SE)^c^	β 95% CI	β (SE)^d^	β 95% CI
Entree, g	239	0.310 (0.272)	−0.218 to 0.843	0.693 (0.364)	−0.013 to 1.403
Fruit, g	230	1.240 (0.348)	0.569 to 1.915	1.083 (0.305)	0.494 to 1.675
Vegetable, g	208	1.685 (0.425)	0.870 to 2.502	1.083 (0.314)	0.482 to 1.684
Beverage, mL^e^	235	0.362 (0.318)	−0.250 to 0.974	1.206 (0.875)	−0.470 to 2.882
Milk, mL	121	0.551 (0.382)	−0.168 to 1.274	1.305 (0.904)	−0.397 to 3.014

^a^ Models included a random effect for participant and fixed effects for seated lunch time, menu, previous menu exposure, mean talking rating, and mean phone use rating.

^b^ Models included a random effect for participant and fixed effects for seated lunch time, menu, previous menu exposure, mean talking rating, mean phone use rating, and preconsumption reference weight or volume.

^c^ The β coefficient was interpreted as the estimated percentage point change in consumption of each tray item for a 1-minute increase in seated lunch time. A positive β coefficient indicated an increase in the percentage consumed.

^d^ The β coefficient was interpreted as the estimated volume or weight change in consumption of each tray item for a 1-minute increase in seated lunch time. A positive β coefficient indicated an increase in the weight or volume consumed.

^e^ Beverage included a selection of milk, water, or both milk and water.

Participants consumed significantly more and wasted significantly less calories (−22.03 kcal; 95% CI, −39.47 to −4.61 kcal), carbohydrates (−3.81 g; 95% CI, −6.20 to −1.42 g), dietary fiber (−0.51 g; 95% CI, −0.81 to −0.19 g), protein (−1.11 g; 95% CI, −2.17 to −0.04 g), iron (−0.20 mg; 95% CI, −0.38 to −0.02 mg), and potassium (−53.49 mg; 95% CI, −84.67 to −22.32 mg) during the 20 minutes, compared with 10 minutes, of seated time (Table 4). No difference was observed in the amount of total fat, vitamin D, and calcium consumed or wasted between the 2 seated lunch conditions. Results of a sensitivity analysis that was restricted to only the 166 tray observations matched to a 10-minute and a 20-minute lunch condition for a menu revealed no differences in conclusions drawn from the full sample.

**Table 4.** Estimated Means of Nutrients Consumed Between 10-Minute and 20-Minute Seated Lunch Times

Nutrient^a^	Mean total nutrients (SD)^b^	Mean (SE)^c^		Contrast (SE)^d^	Contrast 95% CI
		10-min Lunch (n = 126)	20-min Lunch (n = 113)		
Total calories, kcal	421.71 (108.97)	337.73 (18.86)	359.76 (18.46)	−22.03 (8.84)	−39.47 to −4.61
Total fat, g	11.72 (4.13)	9.91 (0.55)	10.27 (0.53)	−0.36 (0.27)	−0.89 to 0.17
Total carbohydrates, g	58.92 (18.25)	45.67 (2.66)	49.48 (2.61)	−3.81 (1.21)	−6.20 to −1.42
Total dietary fiber, g	6.28 (2.28)	4.60 (0.34)	5.11 (0.33)	−0.51 (0.16)	−0.81 to −0.19
Total protein, g	21.88 (7.21)	17.62 (1.11)	18.73 (1.09)	−1.11 (0.54)	−2.17 to −0.04
Vitamin D, μg	1.43 (1.31)	0.95 (0.15)	0.99 (0.14)	−0.04 (0.06)	−0.16 to 0.08
Calcium, mg	364.53 (235.50)	292.52 (22.39)	311.32 (21.92)	−18.81 (11.01)	−40.51 to 2.89
Iron, mg	3.50 (1.44)	2.90 (0.19)	3.10 (0.18)	−0.20 (0.09)	−0.38 to −0.02
Potassium, mg	769.79 (279.39)	564.23 (34.89)	617.72 (34.28)	−53.49 (15.81)	−84.67 to −22.32

^a^ Nutrients represented the total meal, including all entree, fruit, vegetable, and beverage items. Models included a random effect for participant and fixed effects for seated lunch time menu, previous menu exposure, mean talking rating, mean phone use rating, and preconsumption reference nutrients.

^b^ Mean amount of nutrients for the total meal across all seated lunch times.

^c^ Means reported were adjusted for the covariates in the models.

^d^ Contrast was the result of 10-minute minus 20-minute lunch.

## Discussion

In this crossover trial, participants consumed significantly more fruit and vegetables during days with 20 minutes of seated lunch time compared with 10 minutes, whereas no difference was observed in consumption of entrees or beverages. These findings suggest that a shorter seated lunch time differentially affects the rate of fruit and vegetable consumption compared with entrees and beverages. These results also support policies that require 20 minutes of seated lunch time, which could improve diet quality and reduce food waste in children. However, the results also demonstrate the benefit of increasing seated lunch time, even if that time does not meet the recommendations. To our knowledge, this trial was the first to assess the association between seated lunch time constraints and child eating behaviors in a controlled setting using within-person comparisons of food consumption and waste while accounting for the role of social interactions and varying menu items.

In this study, increased vegetable consumption, the meal component least likely to be consumed, occurred during longer lunch times. This finding underscores the need for school meal environments that facilitate protected time for eating vegetables. In a crossover study, Redden et al^27^ concluded that elementary schoolchildren consumed more vegetables when they were offered in a small cup before moving through the lunch line. In another crossover study, Zellner and Cobuzzi^28^ offered fruit only at the end of school lunch instead of during the meal and found that 100% of children at least tried vegetables when fruit was held until later compared with only 40% of children when fruit was not withheld. These 2 previous trials and the current study suggest that children may need protected time to be able to try or consume vegetables.

The results from this study are similar to those from previous observational studies on time for lunch and food consumption. Bergman et al^17^ found that overall food consumption increased with longer lunch periods, and Cohen et al^16^ found that vegetable, entree, and milk (but not fruit) consumption increased with longer lunch time. However, the current study found higher consumption for fruit and vegetable meal components only. The sample in the Cohen et al^16^ study had a comparable age to that of the participants in the current crossover trial; however, participants in the Cohen et al^16^ study were more ethnically diverse (87.6% had Hispanic ethnicity) and had a higher percentage of eligibility for free or reduced-price lunches (88%-94% eligibility).

We compared within-person outcomes, but different responses to the effect of seated lunch time are likely depending on participants’ characteristics. For example, school lunch time constraints are likely to have implications for young children. Fine motor skills, which develop progressively as children get older, are required for self-feeding.^29,30^ Many children are able to feed themselves independently by the time they enter elementary school but have not yet had enough practice to fully master these skills. Thus, younger children may require more time to eat until they further develop self-feeding skills. Because the current study had only 1 participant who was not yet in the third grade, the estimated effect of time constraints on overall lunch consumption may be conservative.

Although statistically significant, the increased vegetable and fruit consumption that occurred during the longer seated lunch condition must also be considered for its practical importance. Consumption of 9.61 more grams of fruit and 8.79 more grams of vegetables may seem like small increases, but these increases in intake suggest that longer seated lunch times improve opportunities for children to try fruits and vegetables. Evidence has shown that repeated tastings are an important step toward food acceptance.^31^

We believe that the current study provides new insights into noneating lunch-time behaviors. Mobile phone use estimates were lower than the estimated time spent talking with other children. Significantly fewer social interactions were observed during the 10-minute seated lunch condition. This finding suggests that longer seated lunch time may present opportunities for children to connect with their peers, which they may not receive during the school day.

This study, along with previous observational studies^16,17^ and evidence that the Healthy, Hunger-Free Kids Act has not increased food waste,^7,8^ suggests that the emphasis for mitigating NSLP food waste should be on other system factors, such as seated lunch time. A standardized policy is needed to ensure that all school-age children get the recommended 20 minutes of seated lunch time. Given the expansive reach of the NSLP, establishing systemic policies around seated lunch time could have important public health implications for school-age children. More research is needed to ascertain whether increased time to eat improves children’s fruit and vegetable consumption at home. Further study is also warranted to examine the effect of seated lunch time constraints on children in kindergarten to second grade and on children from diverse racial/ethnic groups, preferably in a school setting.

### Limitations

This study has several limitations. Summer camp attendance was lower on Thursdays and Fridays, allowing less study participation on those days. Because of logistics, we used only 1 sequence of menus and seated lunch conditions. Randomizing participants to multiple sequences would have allowed for a more traditional crossover study that, by design, controlled for additional effects from the menu and condition schedule. In addition, the logistics of 1 sequence of menus and lunch conditions paired with the convenience sample meant that not every participant had both 10-minute and 20-minute lunch conditions for each of the 5 menus. However, congruent results from the sensitivity analysis that was restricted to observations matched within menus indicated that this limitation likely did not affect the overall conclusions. The findings may not be generalizable to external populations because the sample had limited racial and ethnic diversity, and the data were not collected in an actual school. Although the sample size (38 children and 241 lunch observations) was smaller than the sample sizes in most school-based research, the study’s within-participant design was more efficient than traditional randomized clinical trials.^32^ The sample size of the current study was appropriate for the study design and was adequately powered to detect significant differences in fruit and vegetable consumption.

## Conclusions

This crossover trial provides evidence that a 20-minute seated lunch time is associated with increased fruit and vegetable consumption and decreased waste during lunch in school-age children. These results support a 20-minute seated lunch policy, which could improve diet quality and reduce food waste in children.

## Notes

Supplement 1. Trial Protocol

Supplement 2. Data Sharing Statement

## References

[1] Hales CM, Carroll MD, Fryar CD, Ogden CL. Prevalence of obesity among adults and youth: United States, 2015–2016. NCHS Data Brief No. 288. October 2017. Accessed July 27, 2020. https://www.cdc.gov/nchs/products/databriefs/db288.htm 29155689

[2] US Department of Agriculture. 2015-2020 Dietary Guidelines. Accessed July 27, 2020. https://health.gov/our-work/food-nutrition/2015-2020-dietary-guidelines/guidelines/chapter-2/a-closer-look-at-current-intakes-and-recommended-shifts/

[3] Dietary Guidelines Advisory Committee. Scientific report of the 2020 Dietary Guidelines Advisory Committee: part D: Chapter 1: current intakes of foods, beverages, and nutrients. Accessed July 30, 2020. https://www.dietaryguidelines.gov/sites/default/files/2020-07/PartD_Ch1_CurrIntakes_first-print.pdf

[4] Food and Nutrition Services, USDA. Child nutrition tables. Accessed July 27, 2020. https://www.fns.usda.gov/pd/child-nutrition-tables

[5] Taber DR, Chriqui JF, Powell L, Chaloupka FJ. Association between state laws governing school meal nutrition content and student weight status: implications for new USDA school meal standards. JAMA Pediatr. 2013;167(6):513-519. doi:10.1001/jamapediatrics.2013.399 23567869 PMC4147666

[6] Food and Nutrition Services, USDA. Nutrition standards in the national school lunch and school breakfast programs. 7 CFR 210 and 220. Published January 26, 2012. Accessed July 27, 2020. https://www.federalregister.gov/documents/2012/01/26/2012-1010/nutrition-standards-in-the-national-school-lunch-and-school-breakfast-programs

[7] Cohen JFW, Richardson S, Parker E, Catalano PJ, Rimm EB. Impact of the new U.S. Department of Agriculture school meal standards on food selection, consumption, and waste. Am J Prev Med. 2014;46(4):388-394. doi:10.1016/j.amepre.2013.11.013 24650841 PMC3994463

[8] Schwartz MB, Henderson KE, Read M, Danna N, Ickovics JR. New school meal regulations increase fruit consumption and do not increase total plate waste. Child Obes. 2015;11(3):242-247. doi:10.1089/chi.2015.0019 25734372 PMC4484709

[9] Food and Nutrition Services, USDA. Child nutrition programs: flexibilities for milk, whole grains, and sodium requirements: final rule. Fed Regist. 2018;83(238):63775-63791. 30540150

[10] Buzby JC, Guthrie JF. Plate waste in school nutrition programs: final report to Congress. Accessed July 27, 2020. https://naldc.nal.usda.gov/download/48204/PDF

[11] Frieden TR, Jaffe HW, Stephens JW, Thacker SB, Zaza S; Centers for Disease Control and Prevention (CDC). School health guidelines to promote healthy eating and physical activity. MMWR Recomm Rep. 2011;60(RR-5):1-76. 21918496

[12] Taras H, Luckenbill D, Duncan P, Robinson J, Wheeler L, Woolley S, eds. Health, Mental Health and Safety Guidelines for Schools. American Academy of Pediatrics; 2004.

[13] Hildebrand D, Millburg Ely C, Betts NM, Gates GE. Time to eat school lunch affects elementary students’ nutrient consumption. J Child Nutr Manag. 2018;42(2).

[14] Conklin MT, Lambert LG, Anderson JB. How long does it take students to eat lunch? a summary of three studies. J Child Nutr Manag. 2002;26(2).

[15] US Department of Health and Human Services. Results from the School Health Policies and Practices Study 2016. Accessed July 27, 2020. https://www.cdc.gov/healthyyouth/data/shpps/pdf/shpps-results_2016.pdf#page=63%0Ahttp://www.cdc.gov/healthyyouth/shpps/2012/pdf/shpps-results_2012.pdf

[16] Cohen JFW, Jahn JL, Richardson S, Cluggish SA, Parker E, Rimm EB. Amount of time to eat lunch is associated with children’s selection and consumption of school meal entrée, fruits, vegetables, and milk. J Acad Nutr Diet. 2016;116(1):123-128. doi:10.1016/j.jand.2015.07.019 26372337 PMC4698073

[17] Bergman EA, Buergel NS, Englund TF, Femrite A. The relationship between the length of the lunch period and nutrient consumption in the elementary school lunch setting. J Child Nutr Manag. 2004;28(2).

[18] Food and Nutrition Service, USDA. Breakfast meal pattern: select all three components for a reimbursable meal. Accessed July 30, 2020. https://www.fns.usda.gov/sfsp/meal-patterns

[19] Koo TK, Li MY. A guideline of selecting and reporting intraclass correlation coefficients for reliability research. J Chiropr Med. 2016;15(2):155-163. doi:10.1016/j.jcm.2016.02.012 27330520 PMC4913118

[20] Schakel SF. Maintaining a nutrient database in a changing marketplace: keeping pace with changing food products—a research perspective. J Food Compos Anal. 2001;14(3):315-322. doi:10.1006/jfca.2001.0992

[21] Schakel SF, Buzzard IM, Gebhardt SE. Procedures for estimating nutrient values for food composition databases. J Food Compos Anal. 1997;10(2):102-114. doi:10.1006/jfca.1997.0527

[22] Schakel SF, Sievert YA, Buzzard IM. Sources of data for developing and maintaining a nutrient database. J Am Diet Assoc. 1988;88(10):1268-1271. 3171020

[23] Bates D, Mächler M, Bolker BM, Walker SC. Fitting linear mixed-effects models using lme4. J Stat Softw. 2015;67(1). doi:10.18637/jss.v067.i01

[24] Kuznetsova A, Brockhoff, PB, Christensen RHB. lmerTest package: tests in linear mixed effects models. J Stat Softw. 2017;82(13). doi:10.18637/jss.v082.i13

[25] Lenth R, Singmann H, Love J, Buerkner P, Herve M. Emmeans: estimated marginal means, aka least-square means, version 1.6.0. Accessed May 28, 2021. https://cran.r-project.org/web/packages/emmeans/index.html

[26] GNU Operating System. GNU General Public License. Accessed January 11, 2021. https://www.gnu.org/copyleft/gpl.html

[27] Redden JP, Mann T, Vickers Z, Mykerezi E, Reicks M, Elsbernd S. Serving first in isolation increases vegetable intake among elementary schoolchildren. PLoS One. 2015;10(4):e0121283. doi:10.1371/journal.pone.0121283 25830337 PMC4382151

[28] Zellner DA, Cobuzzi JL. Just dessert: serving fruit as a separate “dessert” course increases vegetable consumption in a school lunch. Food Qual Prefer. 2016;48:195-198. doi:10.1016/j.foodqual.2015.09.013

[29] Gaul D, Issartel J. Fine motor skill proficiency in typically developing children: on or off the maturation track? Hum Mov Sci. 2016;46:78-85. doi:10.1016/j.humov.2015.12.011 26735589

[30] Black MM, Hurley KM. Helping children develop healthy eating habits. Accessed July 30, 2020. https://www.child-encyclopedia.com/child-nutrition/according-experts/helping-children-develop-healthy-eating-habits

[31] Lakkakula A, Geaghan J, Zanovec M, Pierce S, Tuuri G. Repeated taste exposure increases liking for vegetables by low-income elementary school children. Appetite. 2010;55(2):226-231. doi:10.1016/j.appet.2010.06.003 20541572

[32] Harris JE, Raynor HA. Crossover designs in nutrition and dietetics research. J Acad Nutr Diet. 2017;117(7):1023-1030. doi:10.1016/j.jand.2017.03.017 28479137

