# Supplementary material for: Effects of Longer Seated Lunch Time on Food Consumption and Waste in Elementary and Middle School–age Children: A Randomized Clinical Trial
Source: JAMA Netw Open. 2021 Jun 22;4(6):e2114148. doi: 10.1001/jamanetworkopen.2021.14148 (PMC8220493; doi:10.1001/jamanetworkopen.2021.14148)
Supplement: Supplement 1. — Trial Protocol [file jamanetwopen-e2114148-s001.pdf]

PROJECT SUMMARY

Study Title: Time for Lunch: The Impact of Seated Lunch Time Constraints on Child Eating Behaviors

Sponsor: United States Department of Agriculture Hatch Project #ILLU-698-315.

Background and Significance: Recent peer-reviewed observational evidence suggests that school children receive anywhere from 10-33 minutes of seated lunch time and that students with less time to eat discard higher amounts of milk, entree (i.e. protein items), grains, and vegetables. This waste is a missed opportunity for improved nutritional security and healthy habit development. The purpose of this study is to estimate the impact that lunch time constraints have on child food selection, consumption and waste behaviors using a crossover trial design, where seated lunch time is randomly allocated to be either 10 minutes or 20 minutes each day.

STUDY DESIGN

Study Type: Crossover clinical trial (interventional)

Allocation: Randomized

Masking: None (Open Label)

Study Timeframe: June 3, 2019 – June 28, 2019
This study takes place over four consecutive weeks for five days (Monday through Friday) for a total of 20 study days.

Intervention/Treatment: The primary intervention of interest is the time constraints during school meals, randomly assigning either 10 minutes or 20 minutes of seated lunch time to each study day.

Eligibility Criteria:

- Elementary and middle school-aged children attending a university summer camp
- Parent written consent to participate
- Children provide own assent to participate
- Children eat provided camp lunch

Treatment Plan & Randomization: Participants are served school lunches that met National School Lunch Program (NSLP) nutrition standards. Over the course of 20 study days, there are 10 days assigned to each lunch period: a) 10 minutes of seated lunch time and b) 20 minutes of seated lunch time. Five menus were served throughout the course of the study in order to provide variation to the lunches. Lunch time was randomly assigned within each menu so there is a total of two repetitions of each menu and lunch time.

Table 1. *Lunch time assignment and menu pairings* by Study Day*

Week:	Monday	Tuesday	Wednesday	Thursday	Friday
1	June 3<br>A<br>10 minutes	June 4<br>B<br>20 minutes	June 5<br>C<br>10 minutes	June 6<br>B<br>10 minutes	June 7<br>D<br>20 minutes
2	June 10<br>A<br>20 minutes	June 11<br>E<br>10 minutes	June 12<br>C<br>20 minutes	June 13<br>E<br>20 minutes	June 14<br>D<br>10 minutes
3	June 17<br>A<br>10 minutes	June 18<br>B<br>10 minutes	June 19<br>C<br>10 minutes	June 20<br>B<br>20 minutes	June 21<br>D<br>20 minutes
4	June 24<br>A<br>20 minutes	June 25<br>E<br>10 minutes	June 26<br>C<br>20 minutes	June 27<br>E<br>20 minutes	June 28<br>D<br>10 minutes

*There are 5 different menus (labeled A-E). Each menu was served 4 times over the course of the study, consisting of two menu-lunch time dyads occurring during the study period. The lunch time was randomly determined for the first and third occurring menu date, and the alternate lunch time was assigned to the second and fourth occurring date accordingly.

Treatment Arms: Due to logistics, there will be only one sequence of menu and length of lunch throughout the study.

STUDY RECRUITMENT AND STUDY POPULATION

Recruitment: Study participants will be recruited from a university summer camp. All children who sign up for a university summer camp will be invited to participate in the study. Lunch will be provided for free to all children as a benefit, regardless of study participation. There were no additional incentives to participate in the study.

Parents will be sent information about the study, a consent form, and demographic form electronically when signing up for the summer camp. Parents can complete the consent form electronically or bring a hard copy the first day of camp. Parents will also fill out a demographic information form for their child along with the consent form. During the first day of camp, children will provide written assent to participate on the first day of camp each week.

Children can sign up for 1-4 weeks of camp and therefore were not expected to participate in every study day.

METHODS

Menu Plans:
The five menus to serve throughout the study (serving size and NSLP meal component):

Menu A:

- Grilled chicken sandwich on a whole grain bun (2 oz meat, 1-2 oz grain) **OR**
- Whole grain pasta w/ marinara sauce (2 oz grain) and meatballs (2 oz meat)

- Corn (½ C starchy vegetable)
- Broccoli (½ C dark green)
- Grapes (½ C fruit)
- Peaches (½ C fruit)

Menu B:

- Cheese, sausage, or pepperoni pizza (2 oz meat alternative, 2 oz grain) **OR**
- All beef hot dog (2 oz meat/1-2 oz grain)

- Baby carrots (½ C red/orange vegetable)
- Zucchini coins (½ C other vegetable)
- Pineapple (½ C fruit)
- Fresh apple slices (½ C fruit)

Menu C:

- Hamburger/Cheeseburger on whole grain bun (2 oz meat/2 oz grain) **OR**
- Crunchy beef tacos (2 oz meat) with Spanish rice (1 oz grain)

- French fries (½ C starchy vegetable)
- Refried beans (½ C beans/legumes vegetable)
- Pears (½ C fruit)
- Honeydew (½ C fruit)

Menu D:

- Beef with broccoli (2 oz meat and ½ C vegetable) and brown rice (2 oz grain) **OR**
- Sliced turkey and cheese on whole grain sub (2 oz meat/2 oz grain)

- Streamed broccoli (½ C dark green vegetable)
- Steamed cauliflower (½ C other vegetable)
- Mandarin oranges (½ C fruit)
- Peaches (½ C fruit)

Menu E:

- BBQ beef sandwich (2 oz meat) **OR**
- Orange chicken (2 oz meat) and brown rice (2 oz grain)

- Potato wedges (½ C starchy vegetable)
- Sliced red peppers (½ C red/orange vegetable) with pre-portioned ranch
- Strawberries (½ C fruit)
- Apple slices (½ C fruit)

*****_1% unflavored milk or nonfat chocolate milk served with each meal_

Menus will conform to the National School Lunch Program nutrition standards. Food will be prepared by a University chef with assistance from graduate and undergraduate student researchers in a commercial kitchen. All persons involved in preparing meals had a certificate of food handler training. Temperature monitoring and other food safety procedures were strictly followed during food preparation and service.

Menus are designed using MOSIAC software and featured the same foods served to the local school district using the same distributor when possible.

Training of Research Staff:
Research assistants participate in several trainings prior to the start of the study. The first training taught researchers how to complete their respective roles for data collection (e.g., observational data, food waste measurement) and allowed them to practice with each other. The second training consisted of a complete rehearsal of an entire study, including meal production and reference weight collection, participant observation of seated lunch time and social behaviors, and measuring wasted food. During the second training, table layout and placement was refined so that observers could have an unobstructed view of all participants. All research staff also complete CITI ethics trainings and, for research assistants involved in food preparation, food handler training.

Lunchroom Setup and Layout:
Tables are spaced around the lunchroom so that observers are at their own tables with clear sight lines to participants. (Lunchroom layout shown below). Additional observers may stand along the walls (wherever allows clear sightlines) of the room to provide extra observations of participants so that observers reliability can be assessed. Seated observers are provided smaller portions of the same food served to participants to make their observations less noticeable to the participants.

Once participants exit the lunch line, they are permitted to sit at whichever open seat they choose but are not allowed to move chairs.

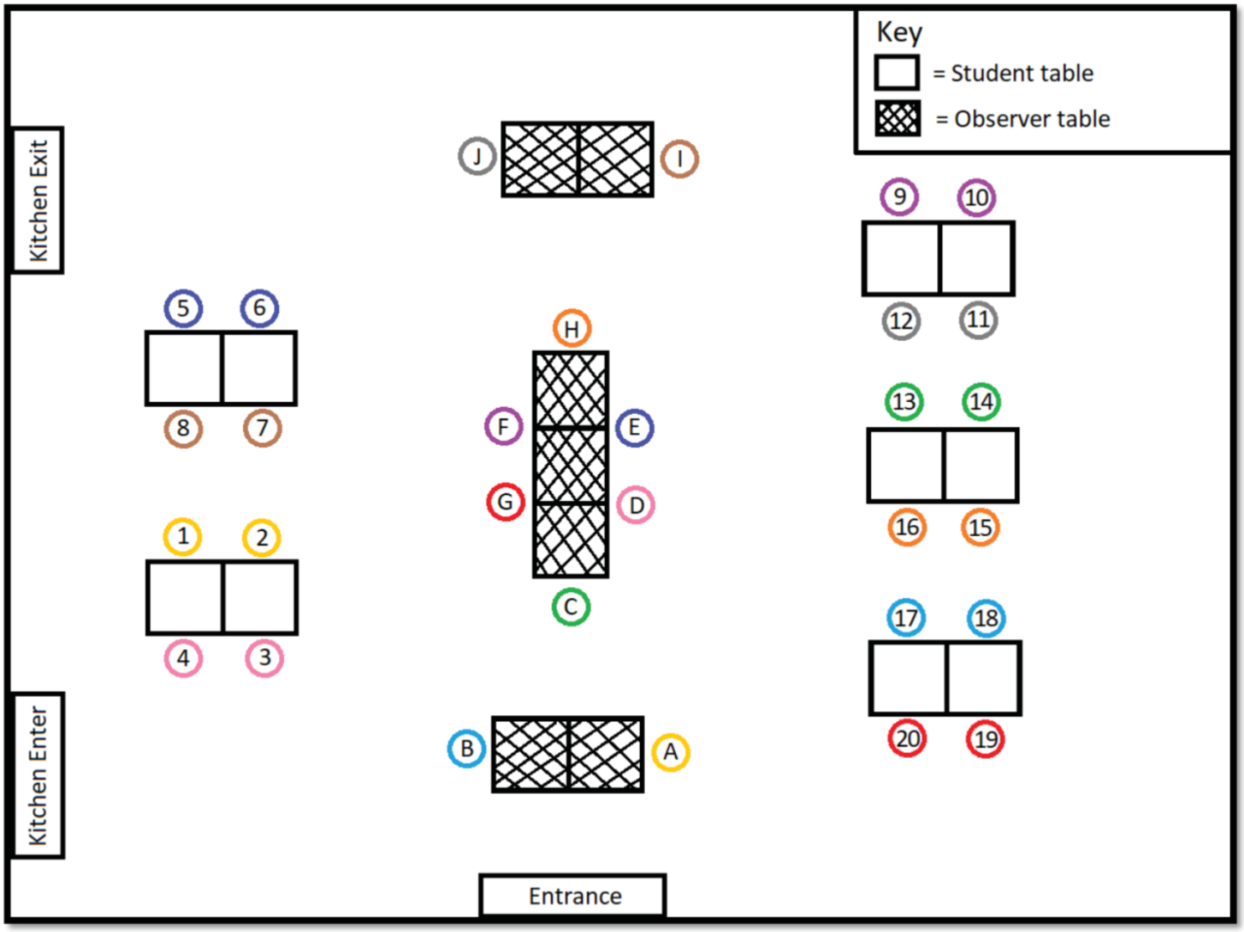

Food Preparation:
For weighing pre-consumption standardized reference weight, five extra servings of each food item will be made, portioned out via standard sized serving utensils, and weighed to the nearest 0.5 grams. The weights of these five samples will be averaged. This average weight will be used as the reference weight.

Foods that are individually packaged (e.g., chicken sandwiches) and have larger weight variation range will be weighed and labeled individually in lieu of reference weights. These items will be marked with a sticker (labeled A-Z) that will be used to track the item's specific weight. A sticker weight log will be used to keep track of stickers and their corresponding weights so that this information will not be visible to participants.

For all foods, inedible portions (cores, peels, etc.) are extracted before the reference weight is measured.

Participants were also offered a healthy snack prepared by research staff at the same time each morning and did not participate in any physical activity before lunch each day. Lunch took place inside the Spice Box, which is a dining space within the department of Food Science and Human Nutrition at the XXXX. At 12:30 pm each day of camp, participants would enter the Spice Box for lunch and immediately be informed if there would 10 or 20 minutes of seated lunch time. Participants were then directed to enter the lunch line, which was located in the adjacent commercial kitchen, very close to the Spice Box entrance.

Lunch Period:
At the start of lunch, participants will form a line to enter the food serving area. Once participants are lined up, a research staff member will make an announcement about the length of seated lunch time for that day. Participants will retrieve their participant ID card and then move through the lunch line.

All food items will be pre-portioned, meaning there will be no option to select portion sizes of items. Children will be instructed to select menu items that qualify as a reimbursable lunch as defined by the National School Lunch Program. Thus, participants are required to select three different components and one must be a fruit or vegetable. (All entree items constituted two meal components: grain and protein). A researcher will check each participant's tray to ensure they have a reimbursable meal after exiting the serving line.

While selecting food items in the lunch line, participants will verbally ask for the food items they want, and research staff will serve the pre-portioned meal items (offer vs. serve format of the National School Lunch Program). Foods will be pre-portioned on small plates, food boats, or bowls. Food components that are hot will be served first due to the arrangement of the buffet equipment.

As participants exit the lunch line, a pre-consumption photo is taken of their selected food items. An ID card with the participant ID number will be included in each tray photograph.

Seated lunch time will officially begin when the last participant makes it through the line and sits down. At this time, a researcher will announce the exact time that lunch ends and will write the time on a poster. The researcher will make an additional announcement when five minutes remain for lunch.

Participants will not be allowed to get seconds on food items, however participants are allowed refills of water. Participants can also get up for condiments and utensils during lunch.

At the end of lunch, a final announcement is made for participants to walk their trays to the corner of the room to collect tray items for weighing.

Data Collection During Lunch Period:

1. Meal component selection: a research staff member takes a photograph of each child's lunch to document selection (which meal component were chosen).

2. Line order: the number order of each child as they leave the survey line, as recorded by a research staff member and documented with a time stamp on the pre-consumption photos.

3. Seat layout: a research staff member documents where each participant sits at the study tables

4. Water refills: a research staff member documents all cups of water children get (unlimited refills were available), recording both the number of cups and the participant ID number.

5. Participant observations: at least one research staff member will observe each participant during lunch time to record information about: time a participant sits down, time a participant stands up, any additional time a participant is not seated in their chair, phone rating and talking during lunch, notes on any food that is spilled or traded, and other notes that may be helpful

during data analysis. *Note: some participants will have multiple observers to provide estimates of inter-rater reliability.*

6. Cover sheet: student researchers will fill out a cover sheet for each study day that includes information about weather and what time lunch started and ended each day.

Data Collected Immediately Following the Lunch Period:

1. Food preference surveys: after handing in their trays, participants will fill out a post-meal survey indicating their preferences on how the food looked and tasted.

2. Food Waste Measurement: A post-consumption photo of each tray will be taken at the end of lunch prior to weighing food items. Each food component (entree, fruit, vegetable) remaining on the tray as food waste will be weighed separately to the nearest 0.5 gram after the lunch period and recorded on weight sheets. Inedible portions of food items will not be weighed (e.g. food bowls, napkins). Beverage waste remaining on the trays will be recorded to the nearest 0.5 mL using a graduated cylinder.

STATISTICAL ANALYSIS

Measures:

Sample Size:
The sample size for this study was fixed based on the summer camp enrollment. A sample size or power calculation was not conducted due to this restraint.

Seated Lunch Time:
Seated lunch time will be measured in two ways in this study. The first measure will be a categorical variable that indicates the randomly assigned seated lunch time: 10 minutes or 20 minutes. There will be a total of 10 10-minute days and 10 20-minute days throughout the study.

The second measure will use the actual time a participant is seated eating lunch. This continuous variable will be calculated using the actual observed seated lunch time. The total seated lunch time is calculated as the time a participant stood up at the end of lunch minus the time a participant sat down at the beginning of lunch. Additional non-seated time recorded on the observer sheet will be subtracted from this lunch time to account for time a participant may have gotten up after initially sitting down.

Inter-rater reliability was assessed for 27.4% of trays during the study that had multiple observers for the same participant. The inter-rater reliability for both the length of lunch and non-seated time had excellent agreement[1] as measured by the intra-class correlation coefficient

- Observed length of lunch: 0.998 (95% CI: 0.997, 0.999)
- Non-seated time: 0.954 (95% CI: 0.888, 0.979)

Food Consumption and Food Waste:

The primary outcomes of interest will be the percent of food items selected, percent of food consumed, and the amount of food wasted. Each outcome will be broken down by meal component: entree, fruit, vegetable, beverage (milk and water), and milk alone.

- Selection: percent of students selecting each meal component
- Percent consumed: $\frac{Reference\ value - amount\ left\ on\ tray}{Reference\ value}$ * 100 *(where the reference value is the pre-consumption weight)*
- Amount wasted: amount of meal component remaining on the tray at the end of lunch, reported in grams (entrée, fruit, and vegetable) or milliliters (beverages)

Nutrient Analysis:
The nutritional profile of all foods served will be determined using the Nutrition Data System for Research. For each food item, nutrients consumed and nutrients wasted will be calculated as follows:

- Nutrients consumed: Percent consumed*reference nutrient *(where the reference nutrient is the pre-consumption value)*
- Nutrients wasted: Percent wasted*reference nutrient *(where the reference nutrient is the pre-consumption value)*

Nutrients will be calculated for individual meal components as well as the total meal. The total meal nutrients will be a sum of the nutrients consumed/wasted from each meal component.

Observed Social Behaviors:
Talking and phone use ratings from the observer sheets will range from a scale of zero to five during the first part of lunch and the last five minutes. An overall rating during lunch will be calculated as the average of the rating for the first part of lunch and the last five minutes.

Inter-rater reliability was assessed for 32.8% of trays during the study that had multiple observers for the same student. The inter-rater reliability for both talking and phone use had excellent agreement[1] as measured by the intra-class correlation coefficient:

- Talking: 0.954 (95% CI: 0.932, 0.967)
- Phone use: 0.967 (95% CI: 0.954, 0.976)

Analysis Plan:

Descriptive statistics: Descriptive statistics will include a count of participants and total trays, frequencies of categorical demographic information, and mean and standard deviation of continuous demographic information.

Selection: Percent of participants who select each food item overall and broken down by 10-minute and 20-minute seated lunch time. Chi square tests can compare the differences in selection distribution between 10-minute and 20-minute seated lunch time .

Model parameters: Mixed effects linear regression models will assess all continuous outcome measures and will include the following:

- Random effects: participants (to control for repeated measures)
- Fixed effects: menu (5 levels), prior menu exposure (4 levels), average talking rating, average phone use rating.

- For amount wasted and all nutrient models: an additional fixed effect of the reference weight/volume or reference nutrient (to control for variability of available food/nutrients pre-consumption)

For models that include the categorical time (10-minute and 20-minute seated lunch time), the estimated marginal means (emmeans) will be used to compare the mean outcome for 10-minute and 20-minute seated lunch time. The contrast between the means (10 minute-mean minus 20-minute mean) will also be reported along with a p-value. Models that used continuous time variable will assess the beta coefficient, t-value, and p-value. Beta coefficients will represent the change in outcome for a one minute increase in seated lunch time.

REFERENCES

1. Koo TK, Li MY. A Guideline of Selecting and Reporting Intraclass Correlation Coefficients for Reliability Research. Journal of Chiropractic Medicine. https://pubmed.ncbi.nlm.nih.gov/27330520/. Published March 31, 2016. Accessed July 29, 2020.
