# Supplementary material for: Effects of Longer Seated Lunch Time on Food Consumption and Waste in Elementary and Middle School–age Children: A Randomized Clinical Trial
Source: JAMA Netw Open. 2021 Jun 22;4(6):e2114148. doi: 10.1001/jamanetworkopen.2021.14148 (PMC8220493; doi:10.1001/jamanetworkopen.2021.14148)
Supplement: Supplement 2. — Data Sharing Statement [file jamanetwopen-e2114148-s002.pdf]

Data Sharing Statement

Burg. Effects of Longer Seated Lunch Time on Food Consumption and Waste in Elementary and Middle School-age Children. *JAMA Netw Open*. Published June 22, 2021. doi:10.1001/jamanetworkopen.2021.14148

Data
Data available: No
